# Right coronary artery deformation and injury following tricuspid valve surgery

**DOI:** 10.3389/fcvm.2022.987993

**Published:** 2022-11-10

**Authors:** Muhammed Gerçek, Hazem Omran, Kai P. Friedrichs, Sabine Bleiziffer, Jan Gummert, Volker Rudolph, Marcus A. Deutsch, Tanja K. Rudolph

**Affiliations:** ^1^Clinic for General and Interventional Cardiology/Angiology, Herz- und Diabeteszentrum NRW, Ruhr-Universität Bochum, Bochum, Germany; ^2^Clinic for Thoracic and Cardiovascular Surgery, Herz- und Diabeteszentrum NRW, Ruhr-Universität Bochum, Bochum, Germany

**Keywords:** right coronary artery deformation, tricuspid valve surgery, tricuspid regurgitation, post-operative coronary angiography, tricuspid valve reconstruction

## Abstract

**Background:**

Due to its close anatomical proximity to the annular plane of the tricuspid valve (TV), the right coronary artery (RCA) is at risk of injury and distortion during surgical and interventional repair. Recently, reversible, non-flow limiting, purse-string like deformations of the RCA following percutaneous TV annuloplasty have been described. In contrast, there are only anecdotal reports on RCA deformation following conventional TV surgery.

**Materials and methods:**

A retrospective analysis of all patients undergoing TV surgery in our hospital between 2009 and 2019 was performed including all patients who received a post-operative coronary angiography (POCA). Angiographic footage was reviewed for RCA affections.

**Results:**

A total of 1,383 patients underwent TV surgery (replacement and repair) for tricuspid regurgitation in our center. TV repair was performed in 1,248 (90.2%) patients and 135 (9.8%) patients underwent isolated TV surgery. Sixty-five patients (4.7%) underwent POCA within 48 h after surgery due to suspected myocardial ischemia, representing the final study population. Mean age was 70.3 ± 11.3 years, 56.3% were female. Mean EuroSCORE II was 9.8 ± 11.6%. Patients with the need for POCA due to suspected myocardial injury suffered from a higher mortality compared to event-free patients over the long-term follow up period (median 2.9 years) regardless of the observed coronary status. RCA affections were observed in 24 (36.9%) patients. A new RCA deformation without flow-impairment or vascular damage was found in 16 (24.6%) of the cases and was managed conservatively. There was no significantly worse outcome observed as compared to patients without RCA affections. Six (9.2%) patients showed an RCA deformation accompanied by subtotal occlusion. A complete RCA-occlusion was observed in 2 (3.1%) patients. Revascularization by percutaneous coronary intervention could be successfully performed in these patients. RCA deformation occurred exclusively after TV repair while no cases were observed after TV replacement.

**Conclusion:**

Right coronary artery deformation without flow-limitation following surgical TV repair is a specific/typical phenomenon which might not impair patients’ outcome and could be managed conservatively in most of the cases. RCA injury indicating further interventional therapy is a rare complication of TV surgery. However, the need for immediate POCA in general appears to be associated with a worsened intermediate-term outcome.

## Introduction

Tricuspid regurgitation is a prevalent valvular heart disease and associated with an increased risk for cardiovascular events and impaired survival if left untreated (1). Surgical treatment is recommended or should be considered in patients with moderate to severe tricuspid regurgitation especially in the context of left-sided valve surgery (2). Due to technical advancements transcatheter tricuspid interventions (TTVI) by means of leaflet approximation, direct annuloplasty, or valve replacement are increasingly considered in patients at high or prohibitive surgical risk and eligible anatomy (3, 4). Conceptually, due to its close anatomical proximity to the annular plane, the right coronary artery (RCA) is at risk to injury and distortion during surgical and interventional procedures for the tricuspid valve (TV) (5). And indeed, recently TTVI-associated distortions of the RCA have been described as relatively frequent phenomenon and are thought to result from the contraction of the often markedly enlarged tricuspid annulus (6). Surprisingly, in the absence of flow-impairment or vascular damage this RCA affection seems to be completely reversible (6).

In the context of tricuspid valve surgery, there are only anecdotal reports on RCA deformations and injuries are anecdotally and based on a single case report (7). Therefore, we herein report our own findings with regard to RCA affections following tricuspid valve surgery using angiographic footage and clinical intermediate-term follow up data.

## Materials and methods

### Study design

A retrospective analysis of patients undergoing tricuspid valve surgery in our center between January 2009 and May 2019 was performed including all patients who received a post-operative coronary angiography due to suspected myocardial ischemia (alterations in electrocardiography, wall motion disorder in transthoracic echocardiography or elevated cardiac enzymes). Fluoroscopic footage was reviewed for RCA deformation and the following therapeutic approach. This study was approved by the local Ethics Committee of the Ruhr University of Bochum (2021-740) and carried out in accordance with the Declaration of Helsinki.

Pre- and post-procedural echocardiographic evaluations were performed by highly qualified medical staff following the recommendations of the American and European Societies and according to the newly proposed tricuspid grading scale (8, 9).

### Surgical techniques

Tricuspid valve surgery was performed using standard techniques. Decisions regarding the use of surgical techniques—including suture placement and the type of prosthetic annuloplasty ring or valve—were at the surgeon’s discretion. Briefly, all procedures were performed with cardiopulmonary bypass and bicaval cannulation. Performance of TV surgery on the arrested or the beating heart was at the discretion of the surgeon. The annuloplasty ring size was usually chosen by measuring the length of the anterior leaflet according to the manufacturer’s instructions. Interrupted non-pledgeted mattress sutures were placed from 9:00 o’clock to 6:00 o’clock (the mid-point of the septal leaflet) around the tricuspid annulus to assure reliable seating of the tricuspid annuloplasty ring.

### Statistical analysis

Statistical analysis was performed using the SPSS-Software (Version 22, IBM Corporation, Armonk, NY, USA). Data are expressed as percentages for categorical variables and as mean ± standard deviation (SD) or median ± interquartile range (ICR) for continuous variables. Continuous variables were compared using Students test and Mann–Whitney U tests as appropriate. Differences between multiple groups with a normal distribution were compared by one-way ANOVA. Differences within groups were analyzed using repeated measures ANOVA or paired *t*-test. If normal distribution was not found, ANOVA on ranks (Kruskal–Wallis) was performed, and Friedmann test and Wilcoxon signed-rank test were used for within-group comparisons. Comparisons of categorical variables between groups were performed by Pearson’s X^2^ test, for expected frequencies <5 by Fisher’s exact test. A time-to-event analysis was performed regarding the study endpoint (all-cause mortality) with the use of Kaplan–Meier survival curves and the log-rank testing. A *p*-value <0.05 was considered significant.

## Results

### Information on tricuspid surgical care

**FIGURE 1:**
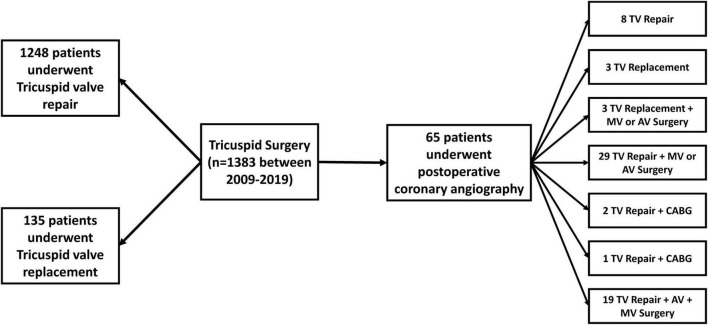
Distribution of surgical procedures in patients who underwent surgery for tricuspid regurgitation. None of the patients who needed post-operative coronary angiography received an aortocoronary bypass on the **right** coronary artery. In three patients bypass grafting was performed on the **left** coronary artery. AV, aortic valve; CABG, coronary artery bypass grafting; MV, mitral valve; TV, tricuspid valve.

Over the period of 11 years (2009–2019), 1,383 patients underwent tricuspid valve surgery for tricuspid regurgitation (308 of them were isolated tricuspid procedures). Of those, 1,248 (90.2%) patients were treated with tricuspid valve repair, while 135 (9.8%) patients underwent valve replacement. In 2.4% of the patients undergoing tricuspid valve repair, the DeVega technique was used. Coronary angiography within 48 h after surgery due to suspected post-operative myocardial ischemia, was performed in 65 (4.7%) patients. Of those, 58 (89.2%) patients underwent tricuspid valve repair (1 in DeVega technique, 57 with ring annuloplasty; 8 isolated and 50 combined procedures with aortocoronary bypass grafting or left-sided surgery) while the remaining seven patients underwent tricuspid valve replacement (three isolated procedures) (Figure 1).

### Patients characteristics

The baseline characteristics and echocardiographic parameters of the total cohort and of the patients with and without RCA affections are provided in Table 1. Patients did not differ significantly with regard to age, sex or comorbidities. Mean age of the patients with and without RCA affections was 69.4 ± 12.5 years and 72.5 ± 6.9 years, respectively. Patients were considered at a higher surgical risk with a mean EuroScore II of 11.7 ± 10.4% and 15.6 ± 16.7%. All patients experienced symptoms of heart failure and were classified as NYHA III to IV despite guideline directed optimal medical therapy. Tricuspid regurgitation was at least graded as severe with a mean vena contracta of 10.1 ± 6.7 mm (10.2 ± 5.4 mm) and an effective regurgitant orifice area of 0.7 ± 0.3 cm^2^ (0.8 ± 0.9 cm^2^). Generally, the right ventricle was dilated in both groups with a right ventricular basal diameter of 51.1 ± 12.3 mm (53.0 ± 10.3 mm) while the right ventricular function was marginally preserved with a fractional area change of 32.6 ± 12.6% (34.5 ± 10.5%) and TAPSE of 19.1 ± 5.0 mm (17.6 ± 5.2 mm).

**TABLE 1:** Baseline characteristics of patients with and without affection of the right coronary artery.

Characteristics	Total (*n* = 1,383)	No RCA-deformation (*n* = 41)	RCA-deformation (*n* = 24)	*P*-value
Age (years)	70.3 ± 11.3	69.4 ± 12.5	72.5 ± 6.9	0.61
Female	778 (56.3%)	19 (46.3%)	9 (37.5%)	0.49
Body mass index (kg/m^2^)	26.4 ± 5.1	27.1 ± 5.9	26.9 ± 4.4	0.82
EuroScore II (%)	9.8 ± 11,6	11.7 ± 10.4	15.6 ± 16.7	0.58
Atrial fibrillation	934 (67.5%)	25 (61%)	13 (54.2%)	0.59
Diabetes mellitus	313 (22.6%)	11 (26.8%)	6 (25%)	0.87
Chronic obstructive pulmonary disease	128 (9.3%)	3 (7.3%)	1 (4.2%)	>0.99
Coronary artery disease	384 (27.8%)	12 (29.3%)	11 (45.8%)	0.18
Prior PCI	185 (13.4%)	3 (7.3%)	6 (25%)	0.07
Ablation procedure	530 (38.3%)	11 (26.8%)	5 (20.8%)	0.59
Extracardiac arteriopathy	79 (5.7%)	3 (7.3%)	1 (4.2%)	>0.99
Stroke	62 (4.5%)	1 (2.4%)	0%	>0.99
Glomerulation filtration rate	59.0 ± 24.0	57.0 ± 24.0	52.0 ± 124.0	0.35
ICD/CRT-device	294 (21.3%)	4 (9.6%)	4 (16.7%)	0.45
Left ventricular ejection fraction (%)	54.0 ± 11.7	52.2 ± 12.6	52.8 ± 11.0	0.79
Tricuspid annular plane systolic excursion (mm)	18.4 ± 4.3	19.1 ± 5.0	17.6 ± 5.2	0.52
Right ventricular basal diameter (mm)	50.5 ± 9.9	51.1 ± 12.3	53.0 ± 10.3	0.38
Fractional area change (%)	32.6 ± 12.6	32.6 ± 12.6	34.5 ± 10.5	0.63
Inferior vena cava diameter (cm)	2.4 ± 0.6	2.6 ± 0.6	2.7 ± 0.5	0.64

Values are given in mean ± SD or *n* (%).

A comparison of the control group with the 65 patients who have received coronary angiography after surgery has been provided in Supplementary Tables 1,2. Baseline characteristics between patients with and without RCA affections did mostly not differ significantly except for the operative risk (Supplementary Tables 1,2).

In total, 21.3% of the patients were under device therapy (pacemaker and/or defibrillator) prior to surgery. The rate of a new device therapy was 20% in our cohort of patients. The incidence of new permanent device therapy was not higher in patients who received post-operative coronary angiography (20.1% vs. 16.9%, *p* = 0.40).

### Right coronary artery findings

**FIGURE 2:**
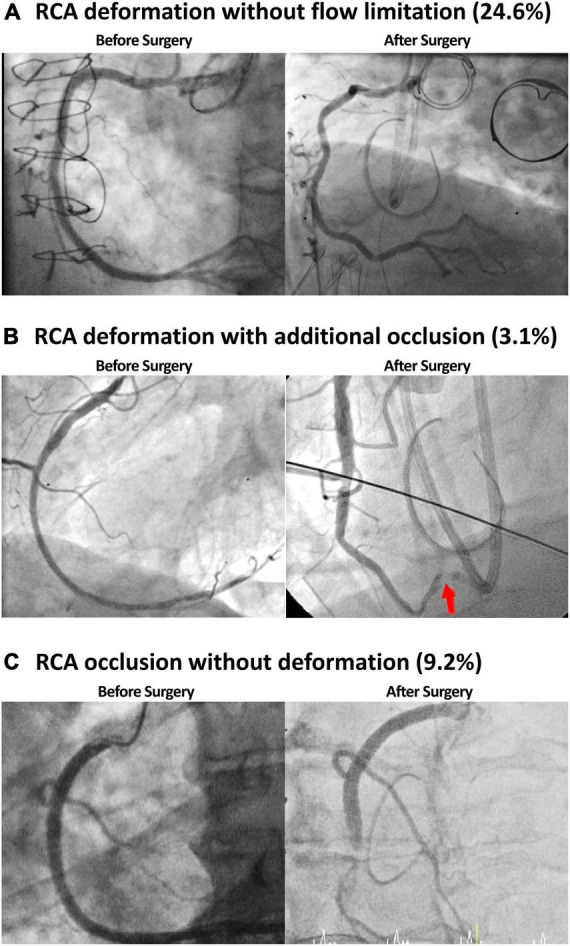
In a third (36.9%) of the patients at least conspicuous right coronary artery (RCA) findings were identified. RCA affection manifested itself as vessel deformation without flow limitation **(A)**, occlusion with additional deformation **(B),** and occlusion without deformation **(C)**.

Baseline angiographic footages of each patients were screened and two patients presented a chronic total RCA occlusion prior surgery. All other patients had no relevant RCA affections before surgery. Twenty-four (36.9%) patients presented with at least conspicuous right coronary artery findings in the post-operative coronary angiography. A new RCA deformation was found in 16 (24.6%) patients without flow-limitations (TIMI 3 flow) and were managed conservatively (Figure 2A). Two (3.1%) patients presented with RCA deformation with additional occlusion (TIMI 0-1 flow) (Figure 2B) while 6 (9.2%) patients showed RCA occlusion without signs of deformation (TIMI 0-1 flow) (Figure 2C). Revascularization by percutaneous coronary intervention could be successfully performed in these patients.

Right coronary artery deformation occurred exclusively after tricuspid valve repair. The rate of RCA deformation did not differ significantly between combined and isolated procedures (*p* = 0.16). Patients with and without RCA affections showed no significant differences in baseline characteristics or echocardiographic features (Tables 1,2). Additionally, tricuspid valve annulus to ring prosthesis diameter did not show a significant difference. The post-operative results regarding residual tricuspid regurgitation did not show a significant difference (Table 2). However, when comparing the shortening of right ventricular parameters (baseline to discharge), patients with post-operative RCA deformation revealed a slightly higher reduction of right ventricular diameters. This, however, did not amount to statistical significance (Table 3).

**TABLE 2:** Pre- and post-operative parameters in patients with and without affection of the right coronary artery.

Characteristics	Total (*n* = 1,383)	No RCA-deformation (*n* = 41)	RCA deformation (*n* = 24)	*P*-value
Duration of surgery (min)	230.0 ± 63.4	275.0 ± 94.0	290.0 ± 60.0	0.23
Bypass time (min)	138.6 ± 103.7	159.7 ± 63.3	176.0 ± 57.9	0.26
Cross clamping time (min)	84.5 ± 41.4	104.3 ± 47.2	115.6 ± 59.6	0.35
Intensive care treatment (h)	214.8 ± 399.7	491.3 ± 486.6	420.7 ± 572.3	0.26
Hospital stay (d)	21.9 ± 17.3	28.0 ± 19.2	26.1 ± 23.9	0.23
Preop. RV midcaval medical diameter (mm)	38.8 ± 9.3	40.8 ± 11.6	39.7 ± 7.6	0.97
Preop. RV enddiastolic area (cm^2^)	25.6 ± 9.3	28.8 ± 17.0	26.5 ± 8.2	0.76
Preop. RV enddsystolic area (cm^2^)	17.3 ± 7.2	18.9 ± 12.8	17.1 ± 7.1	0.93
Preop. right atrial area (cm^2^)	33.2 ± 12.8	25.7 ± 10.4	22.4 ± 9.7	0.44
Preop. right atrial volume (ml)	132.2 ± 85.5	130.5 ± 89.7	128.0 ± 97.3	0.41
Preop. TR vena contracta (mm)	8.4 ± 5.4	10.1 ± 6.7	10.2 ± 5.4	0.67
Preop. TR EROA (cm^2^)	0.8 ± 0.2	0.7 ± 03	0.8 ± 0.9	0.43
Preop. TV tenting height (mm)	9.9 ± 4.1	9.3 ± 4.2	8.9 ± 3.9	0.76
Preop. tenting area (cm^2^)	2.2 ± 1.2	2.1 ± 1.2	2.2 ± 1.3	0.93
Preop. TV annulus diameter (mm)	43.6 ± 8.8	46.6 ± 8.7	49.5 ± 10.8	0.25
Postop. right ventricular basal diameter (mm)	44.8 ± 8.0	44.9 ± 5.1	46.1 ± 7.1	0.35
Postop. RV medical diameter (mm)	36.4 ± 8.1	35.5 ± 5.9	36.7 ± 9.5	0.89
Postop. RV enddiastolic area (cm^2^)	24.6 ± 8.0	23.1 ± 5.9	25.2 ± 9.0	0.76
Postop. RV enddsystolic area (cm^2^)	18.4 ± 7.4	16.7 ± 6.8	18.0 ± 8.7	0.76
Postop. right atrial area (cm^2^)	25.1 ± 8.9	25.7 ± 10.4	22.4 ± 9.7	0.46
Postop. TR vena contracta (mm)	3.9 ± 3.0	4.0 ± 3.2	4.2 ± 3.8	0.74
Postop. TR EROA (cm^2^)	0.2 ± 0.5	0.4 ± 0.6	0.1 ± 0.2	0.67
Postop. TV tenting height (mm)	10.1 ± 4.0	8.1 ± 4.7	7.6 ± 3.3	0.98
Postop. tenting area (cm^2^)	1.7 ± 0.9	1.5 ± 0.8	1.4 ± 0.8	0.83
Postop. TV annulus diameter (mm)	30.1 ± 6.4	38.8 ± 12.3	34.2 ± 10.8	0.40
Postop. tricuspid annular plane systolic excursion (mm)	13.3 ± 3.8	12.2 ± 4.6	16.3 ± 2.3	0.29
Postop. inferior vena cava diameter (cm)	2.2 ± 0.5	2.2 ± 0.5	2.6 ± 0.6	0.17

Values are given in mean ± SD or *n* (%).

**TABLE 3:** Comparison of echocardiographic delta values in patients with RCA affection and patients without RCA alterations.

Imaging parameters Δ (Baseline–Discharge)	RCA affection	No RCA affection	*P*-value
Tricuspid valve annulus diameter (mm)	14.5 ± 11.5	12.6 ± 14.2	0.29
Right ventricular basal diameter (mm)	7.4 ± 8.5	5.4 ± 10.7	0.43
Right atrial area (mm^2^)	130.8 ± 81.0	98.0 ± 111.0	0.77

### Outcome of patients with right coronary artery affections

**FIGURE 3:**
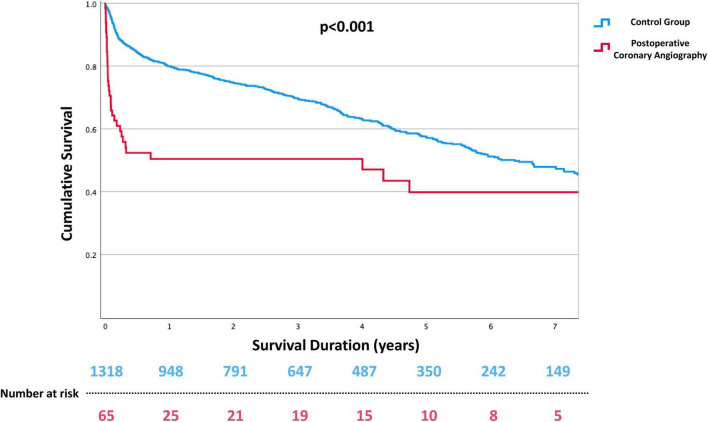
Kaplan–Meyer curves for long-term all-cause mortality after tricuspid valve surgery. Patients requiring immediate post-operative coronary angiography due to suspected myocardial ischemia were shown to have a worse outcome in intermediate-term follow up.

Patients with the need for coronary angiography suffered from a higher unadjusted mortality over the follow up period regardless of the observed coronary status (Figure 3). During median follow-up time of 2.9 (IQR 4.5) years, the unadjusted survival rate for patients with post-operative coronary angiography was 47.7% and 61.4% for no-re-catheterization patients (*p* < 0.001).

**FIGURE 4:**
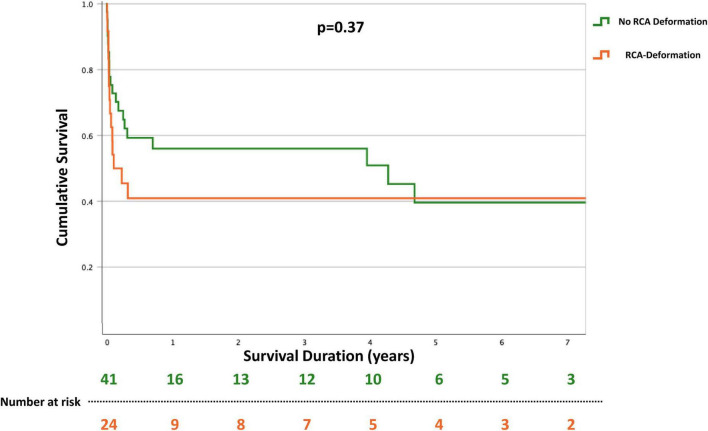
Kaplan–Meyer curves for long-term all-cause mortality in patients with and without right coronary artery (RCA) deformation after tricuspid valve surgery for tricuspid regurgitation. The mortality rate did not differ between patients with and without RCA affections who immediately underwent coronary angiography after surgery.

Of interest, in patients with RCA deformation not resulting in flow limitation no increased mortality could be observed compared to patients who did not show any RCA involvement in the post-surgery re-coronary angiography (41.7% vs. 51.2%, *p* = 0.37) (Figure 4).

## Discussion

This study is the largest assessment of RCA affections following tricuspid valve surgery. The main findings are fourfold. Firstly, long-term outcome in patients who underwent tricuspid valve surgery and had a need for post-operative coronary angiography due to suspected myocardial ischemia or injury is significantly worse. Secondly, post-operative coronary angiography revealed that RCA deformations are not uncommon after tricuspid surgery in patients with post-operative myocardial ischemia and were mainly managed conservatively. Thirdly, RCA deformations without flow limitation do not affect the long-term survival. Finally, only in a few cases (0.6%) of patients who underwent tricuspid surgery interventional therapy on RCA was indicated.

Our data showed that outcome in patients who required post-operative coronary angiography after tricuspid surgery was significantly worse. Post-operative coronary angiography is mostly performed due to hemodynamic instability, post-operative ST-segment elevation, significantly elevated levels of cardiac markers or due to ventricular wall motion disorders in echocardiography indicating myocardial ischemia (10). In some patients, in particular in patients with concomitant left-sided valve procedure, coronary interventions on the left coronary artery were necessary. Even if the coronaries were free from injuries or untreated stenosis or occlusions in these patients, other cardiac and non-cardiac post-operative complications which may have led to the suspicion of a coronary event might explain the higher mortality rate.

Nevertheless, RCA affections during tricuspid surgery were frequently observed in our cohort (36.9% of all patients with coronary angiography). Due to the proximity of the tricuspid annulus and the RCA, injuries of the RCA have been reported as a potential complication in a small case series with patients undergoing surgical tricuspid valve repair (11). However, those patients suffered from RCA occlusion causing hemodynamic or electrical instability. In our study, RCA injury as defined by occlusion necessitating emergency intervention/surgery was identified in 12.3% of the patients who underwent post-operative coronary angiography (0.6% of all patients undergoing TV surgery). Those patients have to be clearly distinguished from patients with RCA deformation without flow limitation. Recently published reports on RCA deformation after interventional tricuspid annuloplasty revealed that RCA deformations without flow limitation recover after a few days (6). Thus, the unreported rate of RCA deformations without flow limitation might be even higher, as it seems not to be clinically harmful (12). Yet, long-term data were still lacking. Our results indicate that even in long-term follow up the patient’s outcome is not negatively affected by RCA deformation without flow limitation. Therefore, our results support the conservative management of these patients with close clinical monitoring and without the need for surgery or intervention in the setting of interventional as well as surgical treatment of tricuspid regurgitation.

Additionally, RCA affections seems to appear noticeably more often after tricuspid repair as no RCA affection was seen in patients with a tricuspid replacement strategy.

There is a slight trend, that the delta values (before surgery minus discharge) of right ventricular and atrial parameters (annulus diameter, basal diameter, and atrial area) are higher in patients with RCA deformation than in patients without. The fact, that RCA affections occur solely after tricuspid valve repair suggests, that the risk for RCA deformation is increasing along increasing reduction size of right ventricular parameters notwithstanding the lack of statistical significance which could possibly be due to a too small sample size.

### Limitation

Several limitations apply to our study. Beside the retrospective design, this study could only search for RCA deformation after tricuspid surgery in patients who required post-operative coronary angiography due to suspected myocardial ischemia. The number of unreported cases might presumably be higher. A possible impact of undetected RCA deformation on patient outcome after tricuspid surgery therefore remains in parts elusive. However, small sample size of patients with post-operative coronary angiography after tricuspid surgery is a shortcoming of our study which might conceal significant effects and correlations.

## Conclusion

Our data suggest that RCA deformation without flow limitation following surgical tricuspid valve repair is a specific/typical phenomenon which might not negatively affect patients’ outcome. The need for immediate post-operative coronary angiography, which is possibly related to unmeasured preoperative and operative risk factors, appears to be associated with a worsened intermediate-term outcome in patients undergoing tricuspid valve surgery.

## Data availability statement

The raw data supporting the conclusions of this article will be made available by the authors, without undue reservation.

## Ethics statement

The studies involving human participants were reviewed and approved by Ethikkommission der Medizinischen Fakultät der Ruhr-Universität Bochum Sitz Ostwestfalen. Written informed consent for participation was not required for this study in accordance with the national legislation and the institutional requirements.

## Author contributions

MG, HO, MD, and TR contributed to the conception and design of the study. MG and HO organized the database and performed the statistical analysis. MG, HO, and TR wrote the first draft of the manuscript. MD and SB wrote sections and critically revised the manuscript. JG and VR supervised the study and critically revised the manuscript. All authors contributed to the article and approved the submitted version.
