# Supplementary material for: Right coronary artery deformation and injury following tricuspid valve surgery
Source: Front Cardiovasc Med. 2022 Nov 10;9:987993. doi: 10.3389/fcvm.2022.987993 (PMC9684246; doi:10.3389/fcvm.2022.987993)
Supplement: Supplementary file 1 [file Data_Sheet_1.docx]

Supplemental Table 1: Baseline characteristics of patients with and without postoperative coronary angiography. Values are given in mean ±SD or n (%).

| **Characteristics** | **Total**  **(n=1318)** | **Postoperative Coronary Angiography (n=65)** | **p-value** |
| --- | --- | --- | --- |
| Age [years] | 70.3 ± 11.3 | 70.6 ± 10.8 | 0.85 |
| Female | 750 (56.9%) | 28 (43.1%) | **0.028** |
| Body mass index [kg/m²] | 26.4 ± 5.1 | 27.0 ± 5.3 | 0.34 |
| EuroScore II [%] | 9.6 ± 11.5 | 13.1 ± 13.1 | **0.018** |
| Atrial fibrillation | 896 (68.0%) | 38 (58.5%) | 0.11 |
| Diabetes mellitus | 296 (22.5%) | 17 (26.2%) | 0.50 |
| Chronic obstructive pulmonary disease | 124 (9.4%) | 4 (6.2%) | 0.38 |
| Coronary artery disease | 361 (27.4%) | 23 (35.4%) | 0.16 |
| Prior PCI | 176 (13.4%) | 9 (13.8%) | 0.51 |
| Ablation procedure | 489 (37.1%) | 16 (24.6%) | **0.041** |
| Extracardiac arteriopathy | 116 (8.8%) | 6 (9.2%) | 0.91 |
| Stroke | 61 (4.6%) | 1 (1.5%) | 0.20 |
| Glomerulation filtration rate | 58.6 ± 24.5 | 55.0 ± 23.7 | 0.26 |
| ICD/CRT-Device | 296 (21.7%) | 8 (12.3%) | 0.07 |
| Left ventricular ejection fraction [%] | 52.4 ± 11.4 | 49.3 ± 12.8 | 0.20 |
| Tricuspid annular plane systolic excursion [mm] | 18.4 ± 4.3 | 18.5 ± 5.0 | 0.90 |
| Right ventricular basal diameter [mm] | 44.7 ± 8.1 | 45.3 ± 5.7 | 0.71 |
| Fractional area change [%] | 32.4 ± 12.5 | 36.4 ± 13.0 | **0.027** |
| Inferior vena cava diameter [cm] | 2.2 ± 0.5 | 2.3 ± 0.6 | 0.47 |

Table 2: Pre- and postoperative parameters in patients with and without postoperative coronary angiography. Values are given in mean ±SD or n (%).

| **Characteristics** | **Total**  **(n=1318)** | **Postoperative Coronary Angiography (n=65)** | **p-value** |
| --- | --- | --- | --- |
| Duration of surgery [min] | 227.7 ± 61.3 | 280.4 ± 82.9 | **<0.001** |
| Bypass time [min] | 137.3 ± 105.1 | 165.7 ± 61.4 | **0.031** |
| Cross clamping time [min] | 83.4 ± 40.5 | 108.5 ± 51.9 | **<0.001** |
| Intensive care treatment [h] | 224.6 ± 389.8 | 465.3 ± 516.6 | **<0.001** |
| Hospital Stay [d] | 21.7 ± 17.1 | 27.3 ± 20.9 | **0.011** |
| Preop. RV midcaval diameter [mm] | 36.5 ± 8.1 | 35.8 ± 7.0 | 0.68 |
| Preop RV enddiastolic area [cm²] | 24.6 ± 8.1 | 23.8 ± 6.9 | 0.57 |
| Preop RV enddsystolic area [cm²] | 18.5 ± 7.4 | 17.1 ± 7.2 | 0.31 |
| Preop. right atrial area [cm²] | 33.1 ± 12.7 | 34.8 ± 13.6 | 0.36 |
| Preop. right atrial volume [ml] | 131.9 ± 85.1 | 138.8 ± 92.3 | 0.57 |
| Preop. TR vena contracta [mm] | 8.3 ± 5.4 | 10.1 ± 6.1 | 0.19 |
| Preop. TR EROA [cm²] | 0.83 ± 2.0 | 0.8 ± 1.2 | 0.84 |
| Preop. TV Tenting height [mm] | 10.0 ± 4.1 | 9.2 ± 4.1 | 0.16 |
| Preop. Tenting area [cm²] | 2.2 ± 1.2 | 2.1 ± 1.2 | 0.85 |
| Preop. TV annulus diameter [mm] | 43.4 ± 8.7 | 47.7 ± 9.5 | **0.001** |
| Postop. Right ventricular basal diameter [mm] | 44.7 ± 8.1 | 45.3 ± 5.7 | 0.71 |
| Postop. RV midcaval diameter [mm] | 36.5 ± 8.1 | 35.8 ± 7.0 | 0.68 |
| Postop. RV enddiastolic area [cm²] | 24.6 ± 8.1 | 23.8 ± 6.9 | 0.31 |
| Postop. RV enddsystolic area [cm²] | 18.5 ± 7.4 | 17.1 ± 7.2 | 0.57 |
| Postop. right atrial area [cm²] | 25.2 ± 8.9 | 24.5 ± 10.1 | 0.72 |
| Postop. TR vena contracta [mm] | 3.9 ± 3.0 | 4.0 ± 3.0 | 0.82 |
| Postop.TR EROA [cm²] | 0.2 ± 0.5 | 0.3 ± 0.5 | 0.44 |
| Postop. TV Tenting height [mm] | 10.2 ± 4.0 | 8.0 ± 4.4 | **0.003** |
| Postop. Tenting area [cm²] | 1.7 ± 0.9 | 1.4 ± 0.8 | 0.17 |
| Postop. TV annulus diameter [mm] | 29.9 ± 6.0 | 37.4 ± 11.9 | **<0.001** |
| Postop. Tricuspid annular plane systolic excursion [mm] | 13.3 ± 3.8 | 13.1 ± 4.5 | 0.83 |
| Postop. Inferior vena cava diameter [cm] | 2.2 ± 0.5 | 2.3 ± 0.6 | 0.47 |
